# Antiviral Effect of Resveratrol in Piglets Infected with Virulent Pseudorabies Virus

**DOI:** 10.3390/v10090457

**Published:** 2018-08-27

**Authors:** Xinghong Zhao, Wenzhi Tong, Xu Song, Renyong Jia, Lixia Li, Yuanfeng Zou, Changliang He, Xiaoxia Liang, Cheng Lv, Bo Jing, Juchun Lin, Lizi Yin, Gang Ye, Guizhou Yue, Yin Wang, Zhongqiong Yin

**Affiliations:** 1 Natural Medicine Research Center, College of Veterinary Medicine, Sichuan Agricultural University, Chengdu 611130, China; zhaoxinghong8@163.com (X.Z.); 13056679601@163.com (W.T.); songx@sicau.edu.cn (X.S.); lilixia905@163.com (L.L.); yuanfengzou@sicau.edu.cn (Y.Z.); lorri190@126.com (C.H.); liangxiaoxia@sicau.edu.cn (X.L.); lvcheng1980@163.com (C.L.); jingbooo@163.com (B.J.); juchunlin@126.com (J.L.); yinlizi@hotmail.com (L.Y.); yegang800206@163.com (G.Y.); 2 Key laboratory of Animal Disease and Human Health of Sichuan Province, Sichuan Agricultural University, Chengdu 611130, China; yaanwangyin@tom.com; 3 College of Science, Sichuan Agricultural University, Ya’an 625014, China; yueguizhou@sicau.edu.cn

**Keywords:** antiviral activity, resveratrol, virulent pseudorabies virus, piglets

## Abstract

Pseudorabies virus (PRV) is one of the most important pathogens of swine, resulting in devastating disease and economic losses worldwide. Nevertheless, there are currently no antiviral drugs available for PRV infection. Resveratrol (Res) was identified to exert its antiviral activity by inhibiting the PRV replication in preliminary investigations. In our previous study, we found that Res has anti-PRV activity in vitro. Here, we show that Res can effectively reduce the mortality and increase the growth performance of PRV-infected piglets. After Res treatment, the viral loads significantly (*p* < 0.001) decreased. Pathological symptoms, particularly inflammation in the brain caused by PRV infection, were significantly (*p* < 0.001) relieved by the effects of Res. In Res-treated groups, higher levels of cytokines in serum, including interferon gama, interleukin 12, tumor necrosis factor-alpha and interferon alpha were observed at 7 days post infection. These results indicated that Res possesses potent inhibitory activity against PRV-infection through inhibiting viral reproduction, alleviating PRV-induced inflammation and enhancing animal immunity, suggesting that Res is expected to be a new alternative control measure for PRV infection.

## 1. Introduction

Pseudorabies virus (PRV; also called Aujeszky’s disease virus or suid herpesvirus type 1), is a member of the *Alphaherpesvirinae* subfamily within the family *Herpesviridae*, and is the causative agent of Aujeszky’s disease (AD), which is one of the most devastating infectious diseases of swine and results in significant economic losses for the swine industry [1,2]. AD is a contagious disease which is characterized by encephalomyelitis, frequently accompanied by inflammation of the upper respiratory tract and lungs [3]. In general, PRV mainly infects pigs at various production phases, such as causing nervous system disorders and high mortality in newborn piglets, respiratory disorders in older pigs, and reproductive failure in sows [1,4,5]. Despite widespread use of the Bartha-K61 vaccine in controlling PRV, AD continues to be one of the most important diseases in pigs in many countries, particularly in regions with dense pig populations, including China [6,7]. Outbreaks of AD in pigs caused by PRV variants happen frequently, even in herds immunized with the Bartha-K61 vaccine. Moreover, new prevalent PRV strains have caused great economic losses to the swine industry in China since 2011 [5,8,9,10,11,12,13].

Resveratrol (3,5,4-trihydroxystilbene, Res), a non-flavonoid polyphenol compound exists widely in several higher plants. Res has been reported to have antiviral activity against a series of viruses either in vitro or in vivo, including herpesviruses [14,15,16], retroviruses [17,18], respiratory syncytial virus [19] and human immunodeficiency virus type 1 [20]. Although Res has been known to have antiviral activity for many years, the use of Res to treat virus infection in relevant virus-host systems has rarely been undertaken.

Previously, we determined the anti-PRV activity of Res for the first time in vitro. The results showed that Res could effectively inhibit virulent PRV replication in vitro [21]. However, little is known about in vivo antiviral activity of Res against PRV. In this study, the anti-PRV activity of Res in piglets infected with virulent PRV was determined in order to develop a new alternative control measure for PRV infection and investigate the antiviral activity of Res in a relevant virus-host system.

## 2. Materials and Methods

### 2.1. Compounds

Resveratrol (Purity of 98%; Sigma, St. Louis, MO, USA) was dissolved in 0.5% carboxymethylcellulose-phosphate-buffered saline (SCMC-PBS) before use.

### 2.2. Virus and Piglets

Virulent PRV (Rong A strain, purchased from China Veterinary Culture Collection Center) was propagated in PK-15 cells. 28-day-old healthy (before the Res administration, the piglets had been investigated for 7 days, with no disease symptoms being observed) piglets were purchased from a remote mountain village (Leshan, China), and no PRV gB-specific antibody were detected through ELISA assay (IDEXX, Westbrook, ME, USA). Piglets were maintained under normal daylight and fed with a standard commercial diet and water ad libitum.

### 2.3. Ethics Statement

All procedures involving animals and their care in this study were approved by the Ethics Committee of Sichuan Agricultural University according to the Regulation of Experimental Animal Management (State Scientific and Technological Commission of the People’s Republic of China, No.2, 1988) and the Interim Measures of Sichuan Province Experimental Animal Management (Science and Technology Bureau of Sichuan, Sichuan, China, No.25, 2013) (the approved permit number is XF2014-18).

### 2.4. Experimental Design

Fifty 35-day-old piglets were randomly divided into five groups. Before infection, the piglets in the Res-treated groups were administered Res through addition into the commercial diet at doses of 30 (Res-H), 10 (Res-M) and 3 (Res-L) mg/kg body weight daily for 7 days. The piglets in the untreated and non-infected groups received only the commercial diet. At 42-days-old, piglets were infected intranasally with 1 mL of 2 × 10^6^ TCID_50_ PRV, except the non-infected group. After infection, the piglets in all of the groups were fed the standard commercial diet. The infected piglets were immediately administered Res solutions orally at doses of 90 (Res-H), 30 (Res-M) and 10 (Res-L) mg/kg body weight twice daily for 21 days, respectively. The dosages of Res before and after infection were based on our previous research: Fu et al., 2018 and Zhao et al., 2017, respectively [21,22]. The piglets in the untreated and non-infected groups were given the same volume of SCMC-PBS. All animals were physically examined daily, and nasal swabs were taken at regular intervals after infection to monitor virus excretion. Serum samples were taken at 0, 7, 14, and 21 days post infection (dpi). Three randomly selected piglets were subjected to necropsy in each group at 7 and 21 dpi. The rearing conditions were based on the Guidelines of the International Committee on Laboratory Animals.

### 2.5. Analysis of Viral Load by Real-Time PCR

The PRV load of piglets was monitored by the real-time fluorescence quantification PCR (FQ-PCR) of nasal swab and brain tissue. The total DNA was isolated from the nasal swabs and brains tissue by TIANamp Swab DNA Kit (Tiangen Biotech, Beijing, China) and Genomic DNA Extraction Kit (TaKaRa, Tokyo, Japan), respectively. The upstream and downstream primers were 5′- ACAAGTTCAAGGCCC ACATCTAC -3′ and 5′- GTCYGTGAAGCGGTTCGTG AT -3′, respectively, which were used to amplify a 95-bp fragment of the glycoprotein B gene of PRV (GenBank accession no. KJ526438). A 17-bp probe (5′- ACGTCATCGTCACGACC -3′) complementary to an internal region between two primers was selected and labelled with carboxyfluorescein at the 5′ end and with carboxytetramethylrhodamine at the -3′ end. The FQ-PCR was analyzed by using SsoAdvancedTM Universal Probes Supermix (BIO-RAD, Hercules, CA, USA) with a Bio-Rad CFX96TM Manager software system according the method described in our previous study [21].

### 2.6. Histopathological Analysis

Histopathological lesions of PRV-infected piglets treated with or without Res were investigated. Heart, liver, kidney, lung, spleen and brain tissues were procured at 7 dpi, preserved in 4% paraformaldehyde, and enclosed in paraffin for subsequent histopathological examination. A 5 μm section of each organ tissue was stained with hematoxylin and eosin. Each section was analyzed under an optical microscope (Nikon eclipse 80i, Tokyo, Japan). Three slides from different parts of each tissue (3 piglets per group) were analyzed. The whole lesions for each tissue were scored by multiplying the degree of severity (0 = no lesions, 1 = mild lesions, 2 = moderate lesions, and 3 = severe lesions) by the extent of lesions (1 = low extent, 2 = intermediate extent, and 3 = large extent) [23].

### 2.7. Serum Cytokines Assay

The concentrations of cytokines in serum, including interferon alpha (IFN-α), interferon gama (IFN-γ), tumor necrosis factor-alpha (TNF-α) and interleukin 12 (IL-12), were detected by using an ELISA kit according to the manufacturer’s instructions (Shanghai Enzyme-Linked Biotechnology Co., Ltd., Shanghai, China).

### 2.8. Statistical Analysis

Data was expressed as the mean ± S.D and the statistical significance of the data was assessed using a two-tailed Student’s *t*-test with GraphPad Prism software 5 (LaJolla, CA, USA). Correlation analyses were evaluated by Pearson r2, ns: *p* > 0.05, * *p* < 0.05 and *p* < 0.001.

## 3. Results

### 3.1. Resveratrol Reduced Mortality and Increased Body Weight Gained by Piglets Infected with Virulent PRV

As shown in Table 1, there were no deaths among groups before 6 dpi, but the piglets began to die in the untreated group at 6 dpi; in contrast, there were no deaths in the Res-treated groups at the same time. The groups treated with Res exhibited a high protection rate (100%, in Res-H and Res-M groups) against PRV infection. However, only six out of ten animals survived in the untreated group.

**Figure 1:**
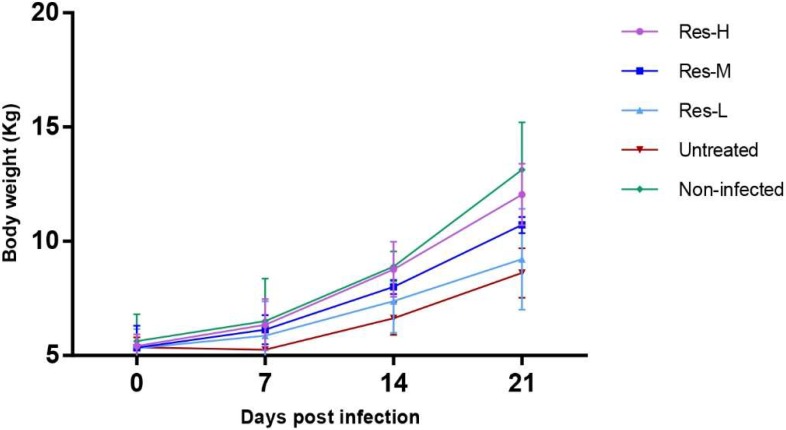
Body weight changes following PRV infection.

Changes in body weight were analyzed over 21 dpi (Figure 1). Compared with the non-infected group, all infected piglets had a reduced gain in body weight. However, compared with the untreated group, the body weight gained increased in the Res-treated groups in a dose-dependent manner.

### 3.2. The Viral Load of Nasal Swab and Brain Were Depressed by Res

**Figure 2:**
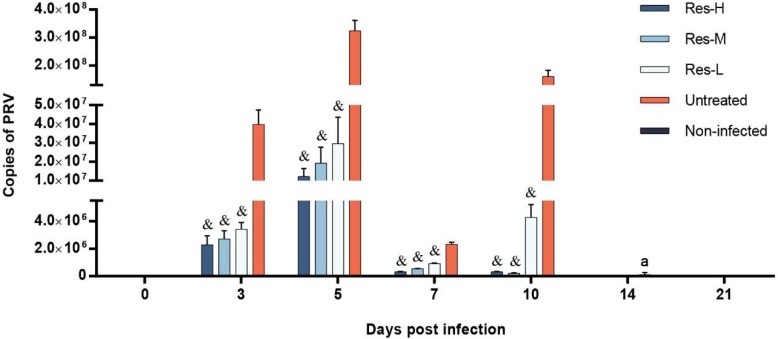
Copies of the PRV genome per nasal swab sample of piglets. Copies of PRV genome in nasal of the piglets were analyzed by FQ-PCR at 0, 3, 5, 7, 10, 14 and 21 dpi (at every selected time point, all of the living piglets were tested, *n* ≥ 3, in each group). There was no PRV genome detected in the non-infected group at any time point and no PRV genome detected at 0, 14 and 21 dpi among the groups. ^a^ There was one piglet detected with PRV in the untreated group at 14 dpi. Correlation analyses were evaluated by Pearson r2, ^&^ *p* < 0.001 vs. untreated group.

The nasal swabs of each group were collected at 0, 3, 5, 7, 10, 14 and 21 dpi, and the viral copies were assayed by FQ-PCR. The results are shown in Figure 2. In the untreated group, virus excretion began to increase rapidly at 3 dpi, while lower viral loads were detected in the Res-treated groups. The viral loads in the Res-treated groups were significantly (*p* < 0.001) lower than that in the untreated group. At 5 dpi, viral loads increased in all infected groups. The Res-treated groups had significantly (*p* < 0.001) lower viral loads compared to the untreated group. At 7 dpi, viral loads in all infected groups decreased, and the viral loads in the Res-treated groups were significantly (*p* < 0.001) lower than those in the untreated group. At 10 dpi, viral loads in Res-H and Res-M groups continually decreased, while the viral loads in Res-L and untreated groups increased; the viral loads in the Res-treated groups were significantly (*p* < 0.001) lower than those in the untreated group. At 14 dpi, there was no PRV genome detected among the groups, except one piglet in the untreated group. There was no PRV genome detected at 21 dpi among any of the groups.

**Figure 3:**
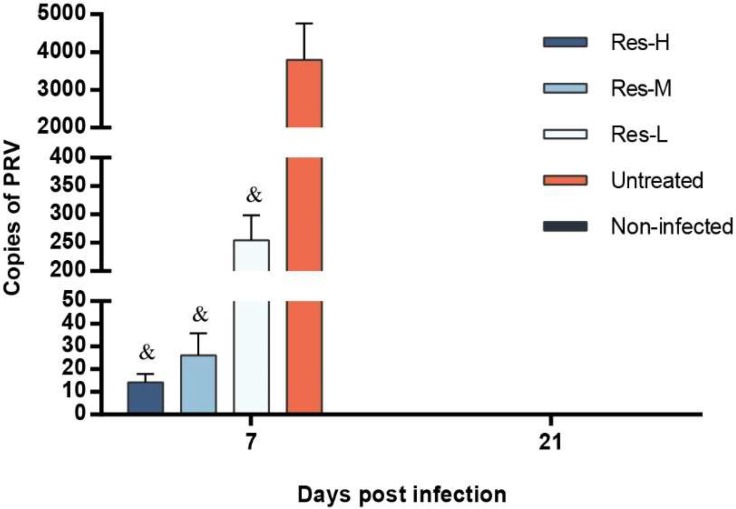
Copies of PRV genome per microgram brain of piglets. Copies of the PRV genome in the brain of the piglets were analyzed by FQ-PCR at 7 and 21 dpi (*n* = 3 in each group). There was no PRV genome detected in the non-infected group at any time point and no PRV genome detected at 21 dpi among the groups. Correlation analyses were evaluated by Pearson r2, ^&^ *p* < 0.001 vs. untreated group.

The brains of each group (three piglets per group) were collected at 7 and 21 dpi, and the viral copies were assayed by FQ-PCR. The results are shown in Figure 3. In the untreated group, viral copies were significantly (*p* < 0.001) higher than those in the Res-treated groups at 7 dpi. There was no PRV genome detected at 21 dpi among any of the groups.

### 3.3. Res Reduced the Pathological Lesions of PRV-Infected Piglets

**Figure 4:**
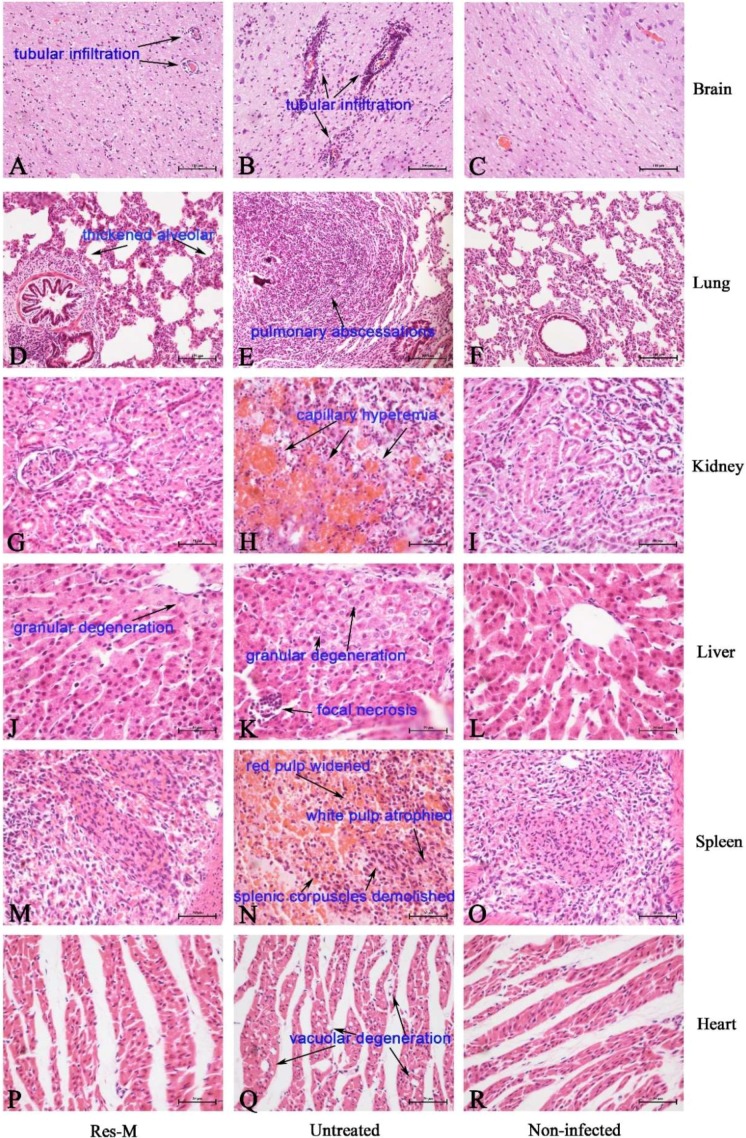
Effect of Res on the microstructures of tissues of piglets at 7 dpi. Panels (**A**–**C**): brain of Res-M, Untreated and Non-infected groups, respectively. Panels (**D**–**F**): lung of Res-M, Untreated and Non-infected groups, respectively. Panels (**G**–**I**): kidney of Res-M, Untreated and Non-infected groups, respectively. Panels (**J**–**L**): liver of Res-M, Untreated and Non-infected groups, respectively. Panels (**M**–**O**): spleen of Res-M, Untreated and Non-infected groups, respectively. Panels (**P**–**R**): heart of Res-M, Untreated and Non-infected groups, respectively. Final magnification ×400, bars = 100 µm.

The PRV-infected piglets of each group (three piglets per group) were dissected at 7 dpi. The different histopathological lesions of brain, liver, lung, kidney, spleen and heart of PRV-infected piglets in the untreated group and Res-M-treated groups are shown in Figure 4. In the brains of the untreated group, a large number of lymphocytes surround the blood vessel, showing tubular infiltration (↑, Figure 4B). There were fewer lymphocytes observed in the brain of the Res-M-treated group (Figure 4A). In the lungs of the untreated group, severe pulmonary abscessations were observed; the alveolar structure disappeared and was infiltrated with many neutrophilic granulocytes (↑, Figure 4E). There were mild lesions observed in the lungs of the Res-M-treated group (Figure 4D): thickened alveolar wall and infiltration with some of the neutrophilic granulocytes. In the kidneys of PRV-infected piglets (Figure 4H), severe capillary hyperemia (↑) was observed. The normal histological structure of the kidney was observed in the Res-M-treated group (Figure 4G). In the livers of the untreated group (Figure 4K), focal necrosis with lymphocytes infiltrated was observed (↑, Figure 4K). Granular degeneration of hepatocytes was observed in the Res-M-treated group (Figure 4J). However, compared with the untreated group, no focal necrosis was observed (Figure 4J). In the spleens of the untreated group (Figure 4N), splenic corpuscles were demolished and had disappeared, red pulp was widened, and white pulp was atrophied (↑). The normal histological structure of spleen was observed in the Res-M-treated group (Figure 4M). Vacuolar degeneration appeared in the hearts of the untreated group (↑, Figure 4Q), while in the Res-M-treated group, the heart basically kept normal histological structure (Figure 4P). These observations were also proven by the lesional score (Table 2).

### 3.4. The Concentrations of Cytokines Were Affected by Res

**Figure 5:**
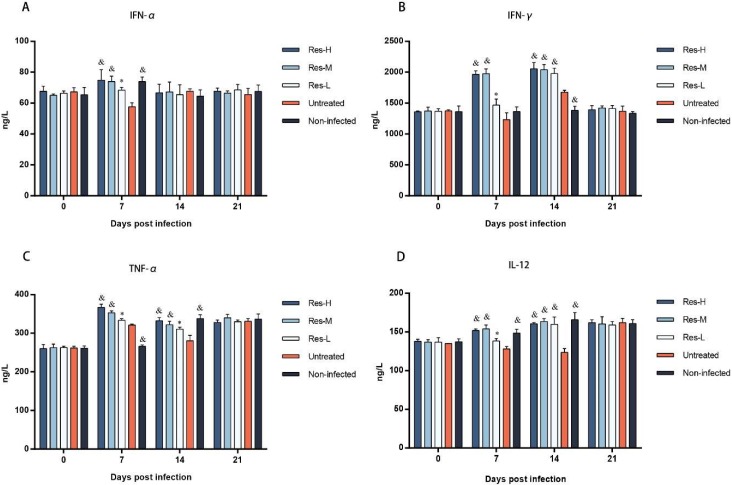
The concentrations of IFN-α (**A**), IFN-γ (**B**), TNF- α (**C**) and IL-12 (**D**) in serum obtained from test piglets. Four cytokines in the serum of the piglets were assayed at 0, 7, 14 and 21 dpi (at each selected time point, all of the living piglets were tested, *n* ≥ 3 in each group). Correlation analyses were evaluated by Pearson r2, ns: *p* > 0.05, * *p* < 0.05 and ^&^ *p* < 0.001 vs. untreated group.

ELISA assays were used to detect the concentrations of IFN-α, IFN-γ, TFN-α and IL-12 in the serum which was separated from the venous blood at 0, 7, 14 and 21 dpi. The results are shown in Figure 5. At 0 dpi, the concentrations of IL-12, TNF-α, IFN-α and IFN-γ showed no significant difference among groups. At 7 dpi, compared with the non-infected group, the concentrations of IL-12 and IFN-α were significantly (*p* < 0.001) decreased in the untreated group, while the decreasing tendency was significantly (*p* < 0.05 or 0.001) inhibited by Res treatment in a dose-dependent manner. Surprisingly, the concentrations of IFN-γ in the untreated group were decreased compared to the non-infected group; however, compared with the non-infected group, the concentration of IFN-γ in Res-treated groups were significantly (*p* < 0.05 or 0.001) increased due to the Res treatment. The concentration of TFN-α was significantly (*p* < 0.001) increased by the infection of PRV. Moreover, compared with the untreated group, Res significantly (*p* < 0.05 or 0.001) increased the concentration of TFN-α. At 14 dpi, compared to the non-infected group, the concentrations of IL-12 and TFN-α were decreased in the untreated group, while the decreasing tendency was significantly (*p* < 0.05 or 0.001) inhibited by Res treatment. The concentration of IFN-γ was significantly (*p* < 0.001) increased due to PRV-infection. Moreover, the concentrations of IFN-γ in Res-treated groups were significantly (*p* < 0.001) higher compared to the untreated group. The concentration of IFN-α showed no significant difference among the groups. At 21 dpi, there were no significant differences of IL-12, TNF-α, IFN-α and IFN-γ levels among the groups.

## 4. Discussion

Although Res has been known to have antiviral activity for many years, the use of Res to treat virus infection in a relevant virus-host system has rarely been done. In our previous study, Res showed potent antiviral activity against virulent duck enteritis virus [14,24]; we also found that Res possessed potent antiviral activity against PRV [21].

This study confirms that Res has a potent antiviral effect in PRV-infected piglets. Addition of Res could reduce mortality rate caused by PRV infection. No piglets died in the Res-H and Res-M groups, and nine out of ten piglets survived in the Res-L-treated group. It should be noted that the body weight gains of the Res-treated groups were higher than that in the untreated group in a dose-dependent manner. Our previous study showed that piglets (without infection) treated with Res were able to gain an insignificant amount of body weight more than control piglets (i.e., non-infected piglets) [22]. Here, we show that Res could help PRV-infected piglets to gain more body weight. These results indicate that Res could be used to reduce the economic losses in PRV-infected piglets by increasing their survival rate and growth performance. These results are consistent with our previous study [14].

Viral load is an important and direct parameter in the evaluation of antiviral effects in vivo [14,23,25,26]. The viral loads of brain tissue and nasal swabs were the most important parameters in the evaluation of virus proliferation and excretion in PRV-infected piglets, respectively [25,26]. In this study, the viral loads were detected by FQ-PCR. The results revealed that Res could significantly inhibit virus excretion, and efficiently reduce virus reproduction. The levels of viral copies in the brain were positively linked to the clinical parameters of infected piglets, which were confirmed by our previous study that Res exerts antiviral activities by inhibiting viral reproduction [14,21,24].

The antiviral effects of Res on PRV-infected piglets were also supported by histopathological observations. In this study, obvious lesions were detected in the brain, lung, kidney, liver, spleen and heart after infection (Figure 4B,E,H,K,N,Q), which were consistent with the previous study [8]. Res significantly decreased the tissues lesions. The results indicated positive therapeutical effects of Res on tissue lesions caused by PRV-infection. Given the high survival ratio and growth performance in the Res-treated groups, we can conclude that Res could effectively inhibit PRV reproduction and suppress the inflammations induced by PRV-infection, and thus decrease the tissue lesions. These results were consistent with our previous reports, which showed that Res could suppress tissue lesions and inflammation [14,27,28].

The immune system plays a key role in protecting the body from foreign pathogens through either innate immunity or acquired immunity. It is well established that innate factors, including IFN-α, IFN-γ, TNF-α and IL-12, play a critical role in inhibiting virus infections; thus, the levels of these cytokines are critical for antiviral immunity [29,30,31,32,33]. In this study, the levels of cytokines (IFN-α, IFN-γ, TNF-α and IL-12) were detected. The results show that the levels of TNF-α in Res-treated groups were significantly higher than that in the untreated group, and the depressed productions of IFN-α, IFN-γ and IL-12 induced by PRV-infection were significantly improved by Res treatment, especially the level of IFN-γ. These results were consistent with our previous reports, which showed that Res could increase the concentrations of IFN-γ in the serum of piglets [22]. Smith et al. reported that IFN-γ-mediated mechanisms play a critical role in the control of and recovery from acute Herpesviridae virus infection [34]. Based on the combination of this information with our results, we can conclude that the higher levels of IFN-γ in the Res-treated groups might be one of the primary reasons for Res having an antiviral effect against virus infection.

In conclusion, Res showed potent antiviral activity on PRV infection in piglets. It was able to decrease the mortality of PRV-infected piglets, enhance growth performance, inhibit viral reproduction, alleviate tissue inflammation and lesions, and improve the levels of cytokines in PRV-infected piglets. The antiviral activity of Res might mainly be attributed to the inhibitory effect on PRV proliferation and immunomodulatory effects of IFN-γ. Resveratrol exhibits potential for PRV control, and further studies should be conducted to evaluate the antiviral activity of Res against infection with other viruses in a relevant virus-host system.

## Figures and Tables

**Table 1:** Resveratrol treatment reduced the mortality of PRV-infected piglets.

Days Post Infection	Survival Rate (%)				
	Res-H	Res-M	Res-L	Untreated	Non-Infected
1	100	100	100	100	100
2	100	100	100	100	100
3	100	100	100	100	100
4	100	100	100	100	100
5	100	100	100	100	100
6	100	100	100	80	100
7 ^a^	100	100	90	60	100

Survival rates of the PRV-infected piglets treated with resveratrol (RV-H, RV-M, RV-L, respectively) and untreated were recorded at 7 dpi (*n* = 10, in each group). ^a^ Date of last death.

**Table 2:** Lesional scores for each group at 7 dpi ^a^.

Group	Res-H	Res-M	Res-L	Untreated
Brain	0.72 ± 0.15 ^&^	0.69 ± 0.21 ^&^	3.90 ± 0.36 *	8.02 ± 0.85
Lung	3.54 ± 0.06 *	3.73 ± 0.21 *	5.74 ± 0.43	7.41 ± 1.43
Kidney	0.65 ± 0.06 ^&^	0.99 ± 0.10 ^&^	4.32 ± 0.31 *	6.39 ± 0.55
Liver	1.84 ± 0.24 *	2.01 ± 0.30 *	4.75 ± 0.23	6.33 ± 1.13
Spleen	N.D.	N.D.	2.28 ± 0.42 *	5.38 ± 0.86
Heart	0.88 ± 0.79 *	0.77 ± 1.33 *	2.48 ± 0.46 *	5.33 ± 0.66

^a^ Lesional scores of each organ were obtained by multiplying the degree of severity (0 = no lesions, 1 = mild lesions, 2 = moderate lesions, and 3 = severe lesions) with the extent of lesions (1 = low extent, 2 = intermediate extent, and 3 = large extent). N.D. means no lesions detected. The values are presented as means ± standard deviation (*n* = 3, in each group). Correlation analyses were evaluated by Pearson r2, ns: *p* > 0.05, * *p* < 0.05 and ^&^ *p* < 0.001 vs. untreated group.
